# Associations of sphingosine-1-phosphate with soluble P-selectin and adverse clinical outcome in patients with cerebral ischemia with and without acetylsalicylic acid treatment

**DOI:** 10.1007/s00210-025-04595-w

**Published:** 2025-10-08

**Authors:** Nils-Ole Gloyer, Eileen Moritz, Laura Schwieren, Ulrike Meyer, Götz Thomalla, Günter Daum, Tim Magnus, Rainer Böger, Chi-un Choe, Bernhard H. Rauch, Edzard Schwedhelm

**Affiliations:** 1 Institute of Clinical Pharmacology and Toxicology, University Medical Center Hamburg-Eppendorf, Hamburg, Germany; 2 German Center for Cardiovascular Research (DZHK e.V.) Partner Site, Hamburg/Kiel/Lübeck, Hamburg, Germany; 3 Department of Pharmacology, University Medicine Greifswald, Greifswald, Germany; 4 Department of Neurology, University Medical Center Hamburg-Eppendorf, Hamburg, Germany; 5 Department of Neurosurgery, University Medical Center Hamburg-Eppendorf, Hamburg, Germany; 6 Department of Pharmacology and Toxicology, University Medicine Oldenburg, Carl von Ossietzky University Oldenburg, Oldenburg, Germany; 7 Department of Vascular Medicine, University Heart and Vascular Center Hamburg-Eppendorf, Hamburg, Germany; 8 Department of Neurology, Klinikum Itzehoe, Itzehoe, Germany

**Keywords:** Acetylsalicylic acid, Aspirin, Antiplatelet drugs, MARK-STROKE, Stroke/prevention

## Abstract

Patients with overt cerebral ischemia are at risk for adverse events after hospital discharge. Sphingosine-1-phosphate is a potent lipid mediator produced and secreted by platelets, which is inhibited via acetylsalicylic acid (ASA). The associations of sphingosine-1-phosphate with biomarkers of platelet activation, i.e., thromboxane B_2_ and soluble P-selectin, as well as with clinical outcome in ischemic stroke or transient ischemic attack patients’ subgroups with and without ASA has not yet been investigated. The bioMARKers in STROKE (MARK-STROKE) cohort is a prospective, single-center, observational study including adult patients with a diagnosis of ischemic stroke or transient ischemic attack. Sphingosine-1-phosphate, thromboxane B_2_, and soluble P-selectin were measured by liquid chromatography-tandem mass spectrometry and ELISA, respectively, to investigate cross-sectional and outcome associations in ASA medication groups. Overall, we included 374 patients (median age 70 (IQR 58; 78) years; sex 242 (64.7%) males; ASA, yes 270 (72.2%)). During the first 365 days of follow-up, we recorded 79 adverse events (death, stroke, myocardial infarction, rehospitalization) in 274 patients with available follow-up. While no statistical differences in neurological and functional deficits were determined by sphingosine-1-phosphate, thromboxane B_2_, and soluble P-selectin in both ASA medication groups, patients without ASA intake and high soluble P-selectin had shorter event-free survival times (high soluble P-selectin 250 (95%CI 205; 296) days; low soluble P-selectin 331 (95%CI 304; 358) days; *p* = 0.005). In addition, adjusted Cox-regression analysis revealed a higher hazard ratio (HR) for an adverse event during follow-up (HR = 3.22 (95%CI 1.41; 7.36); *p* = 0.005). Our findings may indicate the usability of soluble P-selectin in ASA-naive patients with ischemic stroke/transient ischemic attack for risk stratification.

**Supplementary Information:**

The online version contains supplementary material available at 10.1007/s00210-025-04595-w.

## Introduction

Despite recent improvements in the treatment and care of patients, stroke continues to be the third leading cause of death worldwide in 2021 (Collaborators GBDSRF 2024). Approximately one in four persons aged 25 years or older is considered at-risk of experiencing a stroke in later stages of life, leading to direct and indirect healthcare costs equivalent to 1.12% of the world's gross domestic product (Collaborators GBDLRoS et al. 1990; Owolabi et al. 2022). The heterogeneity of stroke pathophysiology is mirrored by the fact that many primary and secondary preventive measures have been introduced in the treatment of stroke patients, such as antiplatelet agents. Drugs like acetylsalicylic acid (ASA, aspirin) inhibit platelet activation and aggregation, reducing the incidence of thromboembolic events in cerebrovascular at-risk patients (Aboyans et al. 2018; Brown et al. 2021).

Platelets are anucleate blood cells that regulate a variety of important vascular functions beyond hemostasis (Gawaz et al. 2005; Stoll and Nieswandt 2019). Thus, aspects of platelet function have been associated with several atherosclerotic conditions, including peripheral or coronary artery disease and myocardial infarction (Trip et al. 1990; Cassar et al. 2003). Moreover, elevated blood markers of platelet activation can be detected during the acute and chronic phases of cerebral ischemia (Marquardt et al. 2002). We previously investigated several blood-based biomarkers, among them sphingosine-1-phosphate, in the bioMARKers in STROKE (MARK-STROKE) cohort including patients with either transient ischemic attack or ischemic stroke (Schwedhelm et al. 2021a). Sphingosine-1-phosphate is a versatile lipid mediator regulating inflammatory processes, which has been discussed recently as a possible biomarker during acute vascular events such as early ischemic stroke (Liu et al. 2020). Identified sources of circulating sphingosine-1-phosphate include activated platelets, which are pharmacologically targeted by ASA via inhibition of platelet activation and consequently reduction of sphingosine-1-phosphate secretion (Polzin et al. 2013; Ulrych et al. 2011), thus underpinning the role of sphingosine-1-phosphate in inflammation due to its sphingosine-1-phosphate receptor 3-mediated mobilization of P-selectin (Nussbaum et al. 2015). Conclusively, other blood-based biomarkers, like thromboxane B_2_, as an established COX1-dependent marker, and soluble P-selectin, may be of particular interest for evaluation of the state of platelet activation in healthy individuals as well as vascular disease patients (Ridker et al. 2001; Spencer et al. 2002; Blann et al. 1995; Nadar et al. 2004). Therefore, we investigated cross-sectional as well as longitudinal associations of sphingosine-1-phosphate in our MARK-STROKE cohort stratified by ASA intake as well as potential underlying confounders including biomarkers of platelet activation.

## Methods

### Ethical statement

The study protocol was approved by the Ethics Committee of the Hamburg Board of Physicians (Approval codes PV4715, PV5979) and conducted in accordance with the Declaration of Helsinki. Enrolled patients or their surrogates provided written informed consent.

### Study design

The bioMARKers in STROKE (MARK-STROKE) cohort is an ongoing investigator-initiated, single-center, prospective, observational study at the University Medical Center Hamburg-Eppendorf, Hamburg, Germany (Schwedhelm et al. 2021a; Schwedhelm et al. 2021b; Schwedhelm et al. 2022; Schwieren et al. 2023). Patients were enrolled consecutively and eligible for inclusion in the study if they had (i) diagnosis of an acute ischemic stroke or transient ischemic attack (TIA) by a board-certified neurologist, were (ii) 18 years or older, and (iii) written informed consent was provided.

The study variables extracted from individual electronic patient records include demographics (age at study inclusion, sex, body mass index), medical history and cardiovascular risk factors (current smoking, arterial hypertension, hyperlipidemia, diabetes mellitus, atrial fibrillation, prior myocardial infarction or stroke), laboratory data (hemoglobin, leucocytes, platelet count, HbA1c, triglycerides, high density lipoprotein (HDL) cholesterol, low density lipoprotein (LDL) cholesterol, creatinine), medication at the time point of blood sample collection (ASA, adenosine diphosphate (ADP) receptor inhibitors, anticoagulants), and clinical characteristics (National Institute of Health Stroke Scale (NIHSS) at admission, modified Rankin Scale (mRS) at admission).

### Outcome variables

For outcome analyses, scores on the mRS and NIHSS were obtained at discharge through in-person, formal, structured interviews and examinations of patients that were performed by qualified assessors. Furthermore, we obtained follow-up data recording adverse events via phone, mail, or email defined as rehospitalization, myocardial infarction, stroke, and death. Events were included until 365 days after the index date.

### Blood collection and laboratory measurements

Plasma blood samples were collected at the time of inclusion by sterile antecubital venipuncture and aspirated into K3 ethylenediaminetetraacetic acid (EDTA) containers (preparation concentration 1.6 mg/mL blood), immediately mixed by inversion, centrifuged at 2000 g and frozen at − 80 °C until further analysis. Similarly, serum blood samples were obtained by aspiration into silicate containers with identical preanalytical preparation procedures. Measurements were performed blinded to clinical data.

Sphingosine-1-phosphate was quantified in serum by liquid chromatography-tandem mass spectrometry method as previously reported (Daum et al. 2020).

Thromboxane B_2_ was measured in serum by ELISA (Cayman Chemical, Ann Arbor, MI, USA) according to the manufacturer’s protocols. In summary, the 96-well plate format was pipetted with blank, non-specific-binding and total activity wells, as well as duplicates of eight steps of the thromboxane B_2_ calibrators, which ranged from 1.6 to 1000 pg/mL. For sample measurement, 50 µL of a 1:20 dilution of serum blood specimen was added to 50 µL of AChE tracer and 50 µL thromboxane B_2_ antiserum, which were then incubated at 4 °C overnight. After incubation, the wells were emptied and rinsed five times with washing buffer. For the development of the plate, 200 µL of Ellman’s reagent was added to each well and incubated in darkness on an orbital shaker for 90 to 120 min. Subsequently, the plate was read at a wavelength of 412 nm. Sample dilutions outside the standard concentrations were repeated with 1:5 or 1:100 dilutions. The sensitivity was 5 pg/mL thromboxane B_2_.

Soluble P-selectin in plasma was determined by a sandwich ELISA (R&D Systems Inc., Minneapolis, MN, USA) according to the manufacturer’s protocols. In brief, 100 µL of standards, samples, and controls was added to the microplate wells, which were precoated with a monoclonal antibody targeting human P-selectin. Standard concentrations ranged from 0.781 to 50 ng/mL. Plasma samples and controls were diluted 1:20 prior to measurement. After adding 100 µL of a polyclonal detection antibody conjugated to horseradish peroxidase, the plate was incubated for 1 h at room temperature and washed three times. Color development was initiated by adding 200 µL of a substrate solution containing hydrogen peroxide and the chromogen, followed by a 15 min incubation in darkness. The reaction was terminated by adding 50 µL of a stop solution and the plate was read at a wavelength of 450 nm.

### Statistics

Continuous variables were reported as medians and interquartile ranges (IQR), whereas categorical variables were presented as absolute numbers and percentages of the total participants. Spearman’s rank correlation test was conducted for bivariate correlation analysis. Mann–Whitney U-test was used for comparing continuous data of two groups. Categorical data were compared using Pearson’s chi-squared test. The median follow-up period was determined using the reverse Kaplan–Meier method. We calculated the risk of an adverse event, such as rehospitalization, myocardial infarction, stroke and death, from the time of inclusion in the study, using univariate and multivariable Cox regression analyses including a crude analysis, and a model with age and sex adjustment. The results of the analyses were presented as hazard ratios (HR) and their corresponding 95% confidence intervals (CI). Event-free survival times, stratified by the defined groups, were presented in the Kaplan–Meier plot. The Kaplan–Meier estimators were given as mean event-free survival times in days and corresponding 95% CIs. Heterogeneity between groups concerning the event-free survival times were determined through the Mantel-Cox test. In the longitudinal analyses, the events until 365 days after inclusion in the study were included into the performed test. IBM SPSS Statistics (version 29.0.1, IBM Corp., Armonk, NY, USA) was used for all statistical analyses. All tests were two-sided. For all cross-sectional analyses, except from baseline characteristics, a global *p*-value below 0.05 was targeted and within analyses local *p*-values below Bonferroni-corrected limits were regarded as significant; otherwise, a *p-*value below 0.05 was regarded significant.

### Data availability

Data extracts from MARK-STROKE that support our findings are available upon reasonable request from the corresponding author.

## Results

### Baseline analyses

From November 2017 until August 2019, a total of 413 consecutive suspected stroke patients passed screening at the Stroke Unit at the Department of Neurology, University Medical Center Hamburg-Eppendorf, Hamburg, of which 374 patients were included. From December 2019 through March 2020, all participants were contacted via telephone, mail, or email for follow-up purposes. During a median follow-up period of 337 (IQR 227; 451) days, we recorded 79 adverse events in 72 patients out of 274 patients with available follow-up. Documented events comprised 18 deaths, 1 non-fatal myocardial infarction, 11 strokes, and 49 rehospitalizations (Supplementary Table 5).

At baseline, patients of the MARK-STROKE cohort had a median age of 70 (IQR 58; 78) years, and 242 (64.7%) were male (Table 1). The three most common vascular risk factors were arterial hypertension (*n* = 272; 72.7%), followed by hyperlipidemia (*n* = 121; 32.4%) and current smoking (*n* = 94; 25.1%). Treatment with ASA was reported in 270 (72.2%) cases. Severity of the neurological deficits, expressed as median NIHSS, was 1 (IQR 0; 3), and the functional status, expressed as median mRS, was 1 (IQR 0; 2).

**Table 1:** Baseline characteristics of total cohort and derivatives. Continuous data are presented in median (interquartile range). Categorical data are presented as absolute numbers (percentage) of participants. Mann–Whitney U or Chi-squared test were used as appropriate. Abbreviations: *ADP*, adenosine diphosphate; *aspirin*, acetylsalicylic acid; *HbA1c*, glycated hemoglobin A1c; *HDL*, high-density lipoprotein; *LDL*, low-density lipoprotein; *NIHSS*, National Institute of Health Stroke Scale; *mRS*, modified Rankin Scale. *p < 0.05, **p < 0.01, ***p < 0.001

Baseline characteristics of patients from the MARK-STROKE cohort			
Characteristics	Aspirin, yes (*n* = 270)	Aspirin, no (*n* = 104)	*p*-value
Demographics			
Age—years	68.0 (57.0; 77.0)	72.0 (61.3; 81.0)	0.051
Male sex—no. (%)	171 (63.3)	71 (68.3)	0.371
Body mass index—kg/m2	25.8 (23.7; 28.4)	25.4 (23.2; 28.4)	0.629
Vascular risk factors			
Current smoking—no. (%)	69 (25.5)	25 (24.0)	0.762
Hypertension—no. (%)	195 (72.2)	77 (74.0)	0.724
Hyperlipidemia—no. (%)	91 (33.7)	30 (28.8)	0.368
Diabetes mellitus—no. (%)	51 (18.9)	13 (12.5)	0.142
Atrial fibrillation—no. (%)	26 (9.6)	59 (56.7)	< 0.001***
Prior myocardial infarction—no. (%)	40 (14.8)	5 (4.8)	< 0.001***
Prior stroke—no. (%)	43 (15.9)	19 (18.3)	0.585
Laboratory			
Hematocrit—%	40.8 (37.3; 44.3)	40.0 (36.6; 42.7)	0.182
Leucocytes—10^9^/L	7.30 (5.88; 8.73)	7.75 (6.13; 9.98)	0.073
Platelets—10^9^/L	238.5 (195.0; 287.3)	232.5 (174.3; 269.5)	0.110
HbA1c—%	5.7 (5.4; 6.1)	5.7 (5.4; 5.9)	0.708
Triglycerides—mg/dL	130.0 (92.5; 179.5)	108.5 (88.0; 151.5)	0.008**
HDL-cholesterol—mg/dL	49.0 (40.0; 60.0)	49.5 (38.0; 59.0)	0.525
LDL-cholesterol—mg/dL	106.0 (77.5; 136.0)	102.0 (74.5; 131.5)	0.445
Creatinine—mg/dL	0.88 (0.74; 1.10)	0.96 (0.82; 1.18)	0.031*
Sphingosine-1-phosphate—µmol/L	1.46 (1.27; 1.73)	1.47 (1.24; 1.68)	0.879
Thromboxane B_2_—pg/mL	416.9 (133.9; 3338)	14,580 (4146; 39,718)	< 0.001***
Soluble P-selectin—ng/mL	125.6 (90.3; 157.4)	93.6 (62.9; 132.6)	< 0.001***
Symptom onset to blood draw—h	47.5 (28.3; 92.0)	42.0 (23.9; 69.0)	0.104
Medication			
Aspirin—no. (%)	270 (100)	0 (0)	
ADP receptor inhibitors—no. (%)	68 (25.2)	8 (7.7)	< 0.001***
Anticoagulants—no. (%)	6 (2.9)	65 (62.5)	< 0.001***
Neurology			
NIHSS—points	1 (0; 3)	2 (0; 4)	0.351
mRS—points	1 (0; 3)	1 (0; 3)	0.887

Assignment of patients according to their medication with ASA deviated two subgroups. Strikingly, there was no difference in sphingosine-1-phosphate levels between patients with and without ASA intake, while there were lower thromboxane B_2_ and higher soluble P-selectin levels in patients with ASA intake compared to patients without ASA intake. As expected, patients with ASA intake presented a significantly lower frequency of atrial fibrillation and a significantly higher frequency of prior myocardial infarction. Furthermore, patients with ASA intake showed significantly higher levels of triglycerides and lower creatinine (Table 1).

Bivariate cross-sectional analyses revealed a robust correlation between sphingosine-1-phosphate and soluble P-selectin in the ASA-treated subgroup (Spearman’s ρ = 0.254, *p* < 0.001) and a weak correlation between sphingosine-1-phosphate and thromboxane B_2_ in the ASA-treated subgroup but not in patients without ASA treatment. Furthermore, sphingosine-1-phosphate in patients with ASA intake was correlated with hematocrit, platelet counts, triglycerides, and LDL-cholesterol (*p* < 0.001 for all) while only being significantly correlated with hematocrit and platelet counts (*p* < 0.01 for both) in patients without ASA intake (Table 2).

**Table 2:** Bivariate correlation analyses of sphingosine-1-phosphate with baseline data. Abbreviations: *aspirin,* acetylsalicylic acid; *HbA1c*, glycated hemoglobin A1c; *HDL*, high-density lipoprotein; *LDL*, low-density lipoprotein. * indicates statistical significance after Bonferroni-correction for multiple comparisons

Cross-sectional correlation analyses of sphingosine-1-phosphate				
	Aspirin, yes (*n* = 270)		Aspirin, no (*n* = 104)	
Variable	Spearman’s ρ	*p*-value	Spearman’s ρ	*p*-value
Age	− 0.127	0.038	− 0.253	0.010
Body mass index	0.018	0.778	0.070	0.497
Hematocrit	0.211	< 0.001*	0.258	0.009
Leucocytes	0.071	0.247	0.221	0.025
Platelets	0.362	< 0.001*	0.296	0.003*
HbA1c	− 0.002	0.978	0.060	0.551
Triglycerides	0.211	< 0.001*	0.162	0.108
HDL-cholesterol	− 0.050	0.415	0.049	0.626
LDL-cholesterol	0.258	< 0.001*	0.203	0.044
Creatinine	− 0.071	0.246	− 0.046	0.649
Soluble P-selectin	0.254	< 0.001*	0.117	0.240
Thromboxane B_2_	0.158	0.041	0.020	0.885

At baseline, participants without functional/neurological deficit (mRS = 0 or NIHSS = 0) versus patients with functional/neurological deficit (mRS > 0 or NIHSS > 0) were equally distributed among the two groups of low or high sphingosine-1-phosphate and soluble P-selectin (Supplementary Table 1).

### Stroke outcomes

At discharge, participants without functional/neurological deficit (mRS = 0 or NIHSS = 0) versus patients with functional/neurological deficit (mRS > 0 or NIHSS > 0) were equally distributed among the two groups with low or high sphingosine-1-phosphate and soluble P-selectin (Supplementary Table 2).

**Fig. 1:**
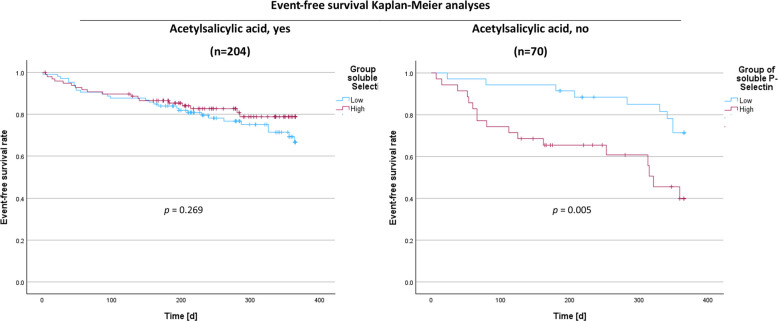
Kaplan–Meier analyses according to reported acetylsalicylic acid intake. Group coding: 1—soluble P-selectin above median, 2—soluble P-selectin below median. Classification limits of plasma soluble P-selectin: median (acetylsalicylic acid, yes) = 125.11 ng/mL, median (acetylsalicylic acid, no) = 91.88 ng/mL

For longitudinal analyses, the Mantel-Cox analyses in both ASA medication groups revealed no differences in event-free survival time between sphingosine-1-phosphate groups; however, Cox-regression analyses in patients with high sphingosine-1-phosphate revealed a significantly lower hazard ratio for an adverse event after adjusting for age and sex in patients with ASA intake (Supplementary Table 3 & 4). For soluble P-selectin, the Mantel-Cox analyses revealed a significantly shorter event-free survival time for patients without ASA intake and high soluble P-selectin levels (Fig. 1). Concomitantly, Cox-regression analyses showed a significantly higher hazard ratio of this group for an adverse event in crude and adjusted analyses (Table 3).

**Table 3:** Cox-regression analyses according to reported acetylsalicylic acid intake. Regression models: A—unadjusted; B—adjusted for age and sex. Abbreviations: *aspirin*, acetylsalicylic acid; *95%CI*, 95% confidence interval; *HR*, hazard ratio. Classification limits of plasma soluble P-selectin: median (acetylsalicylic acid, yes) = 125.11 ng/mL, median (acetylsalicylic acid, no) = 91.88 ng/mL. *p < 0.05, **p < 0.01, ***p < 0.001

Cox-regression analyses for soluble P-selectin						
	Aspirin, yes			Aspirin, no		
High vs. low soluble P-selectin	Model	HR (95%CI)	*p*-value	Model	HR (95%CI)	*p*-value
	A	0.73 (0.40; 1.32)	0.298	A	3.09 (1.36; 7.02)	0.007**
	**B**	0.71 (0.39; 1.29)	0.259	**B**	3.22 (1.41; 7.36)	0.005**

## Discussion and conclusion

In the MARK-STROKE cohort, we found that (i) although sphingosine-1-phosphate levels did not differ between ASA medication groups, (ii) sphingosine-1-phosphate associates with soluble P-selectin in the ASA medication group. Albeit (iii) sphingosine-1-phosphate was not able to distinguish at-risk patients in the group without ASA treatment, patients with high soluble P-selectin presented a shorter event-free survival rate and higher hazard ratio for an adverse event in these patients, exclusively.

Recently, we have shown that serum sphingosine-1-phosphate is inversely associated with severity and outcome after cerebral ischemia (Schwedhelm et al. 2021a). Sphingosine-1-phosphate is produced by and released from platelets upon activation and has been suggested to modulate the platelets’ ability to aggregate in response to certain stimuli (Yatomi et al. 1995; Tani et al. 2005; Urtz et al. 2015; Liu et al. 2021). Even though it is known that the secretion of sphingosine-1-phosphate from human platelets is inhibited by ASA (Ulrych et al. 2011), we observed no differences in sphingosine-1-phosphate between both ASA medication groups but reduced thromboxane B_2_ in the ASA medication group. Conversely, patients with reported ASA intake revealed higher levels of soluble P-selectin, pointing towards other physiological stimuli for secretion of sphingosine-1-phosphate independent from thromboxane mediated platelet activation. Interestingly, there were relevant differences in the associations with sphingosine-1-phosphate according to the ASA use. The significant associations in ASA treated patients with soluble P-selectin, hematocrit, platelet count, triglycerides, and LDL-cholesterol were lessened to a single association with the platelet count in patients without ASA treatment. Thus, underpinning the contribution of other sources to circulatory sphingosine-1-phosphate in patients taking ASA, i.e., red blood cells (Table 2). In line with our previous findings, patients with ASA treatment and high sphingosine-1-phosphate revealed a trend for a lower hazard ratio for an adverse clinical outcome, which became significant after adjustment (Supplementary Table 3).

Soluble P-selectin is a soluble derivative of the membrane-bound P-selectin glycoprotein found on the surfaces of activated platelets and endothelial cells (Stenberg et al. 1985; Bonfanti et al. 1989). Enzymatic cleavage or direct release from the alpha-granules of activated platelets and Weibel-Palade bodies of endothelial cells releases soluble P-selectin into the bloodstream (McEver et al. 1989; Ishiwata et al. 1994; Berger et al. 1998). Elevated levels of soluble P-selectin have been observed in various cardiovascular diseases (CVD), including acute coronary syndrome, ischemic heart disease, and stroke, suggesting its potential use as a biomarker (Blann et al. 1995; Ikeda et al. 1995; Frijns et al. 1997). In atherosclerosis, soluble P-selectin plays a crucial role in the recruitment and adhesion of leukocytes to the endothelium (Woollard et al. 2008). Soluble P-selectin is associated with the formation of monocyte-platelet aggregates, which adhere to the endothelium and facilitate the development and progression of atherosclerotic plaques (Wang et al. 2007; Costa Martins et al. 2004). Additionally, the release of soluble P-selectin promotes a pro-coagulant state, potentially leading to an infarction in downstream arterial vascular territories (Andre et al. 2000). The role of soluble P-selectin in ischemic stroke, the most common type of stroke, is of particular interest and studies have shown that levels of soluble P-selectin are elevated in patients with acute ischemic stroke compared to healthy controls (Frijns et al. 1997). Moreover, soluble P-selectin seemed to predict the adverse event of a stroke after a transient ischemic attack in a large population-based study (Segal et al. 2014).

The concentration of soluble plasma P-selectin measured in our cohort is comparable to other studies on plasma P-selectin in acute and chronic cardio- and cerebrovascular diseases, such as acute myocardial infarction, stroke, ischemic heart diseases, or peripheral artery disease (Blann et al. 1995; Blann et al. 1996; Frijns et al. 1997; Shimomura et al. 1998). Slight differences of soluble P-selectin may be due to the constitution or study design of cohorts (Thom et al. 2004). Notwithstanding the absence of comparability due to the significant disparities in the study population (ischemic and hemorrhagic strokes) and methodologies (severity scales: Glasgow Coma Scale versus mRS/NIHSS), the findings align with those of a previously published study, which also demonstrated an absence of association between soluble P-selectin and short-term outcomes (Bath et al. 1998).

Our findings point towards the need for an improved understanding of the pathophysiological basis and more comprehensive risk assessment of patients with cerebral ischemia. Patients with high soluble P-selectin plasma levels and without an antiplatelet medication such as ASA may be at an elevated risk for an adverse clinical outcome. These patients may benefit from an optimized follow-up and medical interventions aimed at risk reduction. In agreement with this notion, experimental data show an association of high soluble P-selectin in plasma with poorer stroke outcome, i.e., larger infarction volumes and increased blood–brain barrier permeability, even suggesting soluble P-selectin a potential target option for focal cerebral ischemia (Kisucka et al. 2009).

There are several potential limitations that may have affected the results of our study. Firstly, the study protocol primarily allowed for the inclusion of patients who were able to give their consent independently. As a result, patients with severe neurological deficits who were not able to give informed consent may have been under-represented in our study cohort, thus confining the generalizability. However, it should be noted that about one in two hospitalized stroke patients presents with a NIHSS between zero and four; therefore, our presented study is providing insights into to a clinically relevant patient population (Saber and Saver 2020; Reeves et al. 2013). The characteristics of the single university study center in a metropolitan city may contribute to potential selection bias (Hammond et al. 2020). A limited study power could be linked to the small sample size and short duration of follow-up, as demonstrated by relatively wide 95% confidence intervals and a low number of occurrences of deaths, strokes, or myocardial infarctions during the follow-up period, which may restrict the ability to identify a significant association with relevant endpoints. Furthermore, the reasons for rehospitalization were not documented; thus, non-causal associations cannot be ruled out. Finally, the presence of multiple medical conditions in patients with evident cardiovascular diseases and their resulting treatments could increase the likelihood of medication bias. Additionally, the direct verification of soluble P-selectin as a marker for platelet activation through flow cytometry or light transmission aggregometry was not feasible due to the use of frozen plasma samples.

In conclusion, increased plasma soluble P-selectin levels in patients with ischemic stroke or transient ischemic attack without ASA treatment are associated with a higher risk of adverse clinical outcomes in longitudinal analyses. Therefore, plasma soluble P-selectin might be an additional prognostic biomarker of interest for the identification of patients at risk, in addition to the conventional risk assessment in patients with cerebral ischemia.

## Supplementary Information

Below is the link to the electronic supplementary material.

Supplementary file1 (DOCX 35 KB)

Supplementary file2 (DOCX 43 KB)

## Data Availability

Data extracts from MARK-STROKE that support our findings are available upon reasonable request from the corresponding author.
