# Correction to: Associations of sphingosine‑1‑phosphate with soluble P‑selectin and adverse clinical outcome in patients with cerebral ischemia with and without acetylsalicylic acid treatment

**DOI:** 10.1007/s00210-026-05049-7

**Published:** 2026-02-06

**Authors:** Nils‑Ole Gloyer, Eileen Moritz, Laura Schwieren, Ulrike Meyer, Götz Thomalla, Günter Daum, Tim Magnus, Rainer Böger, Chi‑un Choe, Bernhard H. Rauch, Edzard Schwedhelm

**Affiliations:** 1 Institute of Clinical Pharmacology and Toxicology, University Medical Center Hamburg-Eppendorf, Hamburg, Germany; 2 German Center for Cardiovascular Research (DZHK E.V.) Partner Site, Hamburg/Kiel/Lubeck, Germany; 3 Department of Pharmacology, University Medicine Greifswald, Greifswald, Germany; 4 Department of Neurology, University Medical Center Hamburg-Eppendorf, Hamburg, Germany; 5 Department of Neurosurgery, University Medical Center Hamburg-Eppendorf, Hamburg, Germany; 6 Department of Pharmacology and Toxicology, University Medicine Oldenburg, Carl Von Ossietzky University Oldenburg, Oldenburg, Germany; 7 Department of Vascular Medicine, University Heart and Vascular Center Hamburg-Eppendorf, Hamburg, Germany; 8 Department of Neurology, Klinikum Itzehoe, Itzehoe, Germany

**Correction to: Naunyn–Schmiedeberg's Archives of Pharmacology**

10.1007/s00210-025-04595-w

The original version of this article unfortunately contained a mistake.

There is a mistake in Fig. 1 of the original article. The graphs of Fig. 1 have been swapped wherein the p-value of the left part of the figure belongs to the right part and vice versa.

The correct figure should have appeared as shown below.
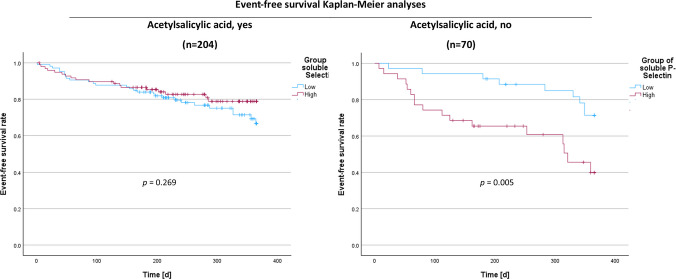


The original article has been corrected.

