# Supplementary material for: Associations of sphingosine-1-phosphate with soluble P-selectin and adverse clinical outcome in patients with cerebral ischemia with and without acetylsalicylic acid treatment
Source: Naunyn Schmiedebergs Arch Pharmacol. 2025 Oct 8;399(3):3743–50. doi: 10.1007/s00210-025-04595-w (PMC12935708; doi:10.1007/s00210-025-04595-w)
Supplement: Supplementary file 1 — Supplementary file1 (DOCX 35 KB) [file 210_2025_4595_MOESM1_ESM.docx]

7.0 SUPPLEMENTARY DATA

| **Neurological and functional deficit at admission for sphingosine-1-phosphate** | | | | |
| --- | --- | --- | --- | --- |
|  | **Acetylsalicylic acid, yes** | | **Acetylsalicylic acid, no** | |
|  | **NIHSS** | | **NIHSS** | |
|  | **0** | **1-42** | **0** | **1-42** |
| **Low sphingosine-1-phosphate** | 41 | 93 | 16 | 35 |
| **High sphingosine-1-phosphate** | 46 | 88 | 17 | 34 |
|  | **mRS** | | **mRS** | |
|  | **0** | **1-6** | **0** | **1-6** |
| **Low sphingosine-1-phosphate** | 34 | 100 | 17 | 34 |
| **High sphingosine-1-phosphate** | 40 | 94 | 15 | 36 |
| **Neurological and functional deficit at admission for soluble P-selectin** | | | | |
|  | **Acetylsalicylic acid, yes** | | **Acetylsalicylic acid, no** | |
|  | **NIHSS** | | **NIHSS** | |
|  | **0** | **1-42** | **0** | **1-42** |
| **Low soluble P-selectin** | 41 | 94 | 17 | 35 |
| **High soluble P-selectin** | 46 | 89 | 16 | 36 |
|  | **mRS** | | **mRS** | |
|  | **0** | **1-6** | **0** | **1-6** |
| **Low soluble P-selectin** | 36 | 99 | 16 | 36 |
| **High soluble P-selectin** | 38 | 97 | 16 | 36 |

Supplementary Table 1: Categorical analyses of neurological and functional deficit at hospital admission. Data is presented as absolute numbers of participants. Abbreviations: NIHSS – National Institute of Health Stroke Scale, mRS – modified Rankin’ Scale. Classification limits of serum sphingosine-1-phosphate: median (acetylsalicylic acid, yes) = 1.458 µmol/L, median (acetylsalicylic acid, no) = 1.471 µmol/L. Classification limits of plasma soluble P-selectin: median (acetylsalicylic acid, yes) = 125.11 ng/mL, median (acetylsalicylic acid, no) = 91.88 ng/mL. No statistically significant difference in distribution of participants.

| **Stroke outcome analyses for sphingosine-1-phosphate** | | | | |
| --- | --- | --- | --- | --- |
|  | **Acetylsalicylic acid, yes** | | **Acetylsalicylic acid, no** | |
|  | **NIHSS** | | **NIHSS** | |
|  | **0** | **1-42** | **0** | **1-42** |
| **Low sphingosine-1-phosphate** | 66 | 68 | 28 | 23 |
| **High sphingosine-1-phosphate** | 74 | 60 | 23 | 28 |
|  | **mRS** | | **mRS** | |
|  | **0** | **1-6** | **0** | **1-6** |
| **Low sphingosine-1-phosphate** | 60 | 74 | 28 | 23 |
| **High sphingosine-1-phosphate** | 68 | 66 | 21 | 30 |
| **Stroke outcome analyses for soluble P-selectin** | | | | |
|  | **Acetylsalicylic acid, yes** | | **Acetylsalicylic acid, no** | |
|  | **NIHSS** | | **NIHSS** | |
|  | **0** | **1-42** | **0** | **1-42** |
| **Low soluble P-selectin** | 66 | 69 | 28 | 24 |
| **High soluble P-selectin** | 75 | 60 | 25 | 27 |
|  | **mRS** | | **mRS** | |
|  | **0** | **1-6** | **0** | **1-6** |
| **Low soluble P-selectin** | 60 | 75 | 27 | 25 |
| **High soluble P-selectin** | 69 | 66 | 24 | 28 |

Supplementary Table 2: Categorical analyses of short-term stroke outcome at timepoint of discharge from hospital. Data is presented as absolute numbers of participants. Abbreviations: NIHSS – National Institute of Health Stroke Scale, mRS – modified Rankin’ Scale. Classification limits of serum sphingosine-1-phosphate: median (acetylsalicylic acid, yes) = 1.458 µmol/L, median (acetylsalicylic acid, no) = 1.471 µmol/L. Classification limits of plasma soluble P-selectin: median (acetylsalicylic acid, yes) = 125.11 ng/mL, median (acetylsalicylic acid, no) = 91.88 ng/mL. No statistically significant difference in distribution of participants.

| **Mantel-Cox analyses for sphingosine-1-phosphate** | | |
| --- | --- | --- |
|  | **Acetylsalicylic acid, yes** | **Acetylsalicylic acid, no** |
|  | **Mean Estimator (95%CI)** | **Mean Estimator (95%CI)** |
| **Low sphingosine-1-phosphate** | 302.3 (280.7; 324.0) days | 312.8 (277.9; 347.6) days |
| **High sphingosine-1-phosphate** | 319.2 (297.9; 340.4) days | 267.0 (222.2; 311.7) days |
| **P value** | 0.057 | 0.143 |
| **Mantel-Cox analyses for soluble P-selectin** | | |
|  | **Acetylsalicylic acid, yes** | **Acetylsalicylic acid, no** |
|  | **Mean Estimator (95%CI)** | **Mean Estimator (95%CI)** |
| **Low soluble P-selectin** | 305.5 (284.3; 326.7) days | 331.1 (304.0; 358.1) days |
| **High soluble P-selectin** | 314.5 (292.7; 336.3) days | 250.3 (204.6; 295.9) days |
| **P value** | 0.296 | 0.005** |

Supplementary Table 3: Kaplan-Meier Estimators according to reported acetylsalicylic acid intake. Abbreviations: 95%CI – 95% confidence interval. Classification limits of plasma soluble P-selectin: median (acetylsalicylic acid, yes) = 125.11 ng/mL, median acetylsalicylic acid, no) = 91.88 ng/mL. Classification limits of serum sphingosine-1-phosphate: median (acetylsalicylic acid, yes) = 1.458 µmol/L, median (acetylsalicylic acid, no) = 1.471 µmol/L. *p<0.05, **p<0.01, ***p<0.001.

| **Cox-regression analyses for sphingosine-1-phosphate** | | | | | | |
| --- | --- | --- | --- | --- | --- | --- |
|  | **Acetylsalicylic acid, yes** | | | **Acetylsalicylic acid, no** | | |
| **High vs Low sphingosine-1-phosphate** | **Model** | **HR (95%CI)** | **P value** | **Model** | **HR (95%CI)** | **P value** |
|  | **A** | 0.56  (0.30; 1.03) | 0.061 | **A** | 1.79  (0.81; 3.94) | 0.150 |
|  | **B** | 0.53  (0.28; 0.98) | 0.044* | **B** | 1.91  (0.85; 4.31) | 0.118 |

Supplementary Table 4: Cox-regression analyses according to reported acetylsalicylic acid intake. Regression models: A – unadjusted; B – adjusted for age and sex; Abbreviations: 95%CI – 95% confidence interval, HR – Hazard ration. Classification limits of serum sphingosine-1-phosphate: median (acetylsalicylic acid, yes) = 1.458 µmol/L, median (acetylsalicylic acid, no) = 1.471 µmol/L. *p<0.05, **p<0.01, ***p<0.001.

| **Baseline characteristics of patients from the MARK-STROKE cohort with follow-up** | | | |
| --- | --- | --- | --- |
| **Characteristics** | **Acetylsalicylic acid, yes**  **(n=204)** | **Acetylsalicylic acid, no**  **(n=70)** | **P value** |
| **Demographics** |  |  |  |
| Age – years | 70.0 (58.25; 78.0) | 73.0 (64.75; 81.0) | 0.088 |
| Male sex – no. (%) | 125 (61.3) | 46 (65.7) | 0.508 |
| Body mass index – kg/m² | 25.71 (23.62; 27.66) | 25.14 (22.64; 27.78) | 0.517 |
| **Vascular risk factors** |  |  |  |
| Current smoking – no. (%) | 46 (22.5) | 17 (24.3) | 0.766 |
| Hypertension – no. (%) | 146 (71.6) | 51 (72.9) | 0.836 |
| Hyperlipidemia – no. (%) | 67 (32.8) | 20 (28.6) | 0.508 |
| Diabetes – no. (%) | 33 (16.2) | 9 (12.9) | 0.506 |
| Atrial fibrillation – no. (%) | 19 (9.3) | 39 (55.7) | <0.001*** |
| Prior myocardial infarction – no. (%) | 26 (12.4) | 3 (4.3) | 0.047* |
| Prior stroke – no. (%) | 25 (12.3) | 12 (17.1) | 0.302 |
| **Laboratory** |  |  |  |
| Hemoglobin – mg/dL | 13.65 (12.5; 14.7) | 13.45 (12.4; 14.53) | 0.400 |
| Leucocytes – 10^9^/L | 7.35 (5.9; 8.98) | 7.75 (6.18; 9.93) | 0.201 |
| Platelets – 10^9^/L | 242 (198; 281) | 228.5 (174.75; 266.25) | 0.035* |
| HbA1c – % | 5.6 (5.4; 6.1) | 5.7 (5.48; 5.9) | 0.840 |
| Triglycerides – mg/dL | 125.0 (91.0; 177.0) | 107.0 (80.0; 154.0) | 0.015* |
| HDL-cholesterol – mg/dL | 49.0 (39.75; 62.0) | 50.0 (39.0; 60.0) | 0.955 |
| LDL-cholesterol – mg/dL | 106.0 (80.0; 135.5) | 105.0 (77.5; 133.5) | 0.866 |
| Creatinine – mg/dL | 0.88 (0.74; 1.1) | 0.92 (0.8125; 1.20) | 0.148 |
| Sphingosine-1-phosphate – µmol/L | 1.458 (1.296; 1.709) | 1.469 (1.236; 1.619) | 0.834 |
| Soluble P-selectin – ng/mL | 123.26 (86.57; 156.69) | 93.63 (62.98; 130.88) | 0.002** |
| **Medication** |  |  |  |
| Acetylsalicylic acid – no. (%) | 204 (100) | 0 (0) |  |
| ADP receptor inhibitors – no. (%) | 46 (22.5) | 6 (8.6) | 0.010* |
| Anticoagulants – no. (%) | 2 (0.9) | 40 (57.1) | <0.001*** |
| **Neurology** |  |  |  |
| NIHSS – points | 1 (0; 3) | 2 (0; 4) | 0.425 |
| mRS – points | 1 (0; 2) | 1 (0; 3) | 0.896 |

Supplementary Table 5: Baseline characteristics of follow-up cohort and derivatives. Continuous data is presented in median (interquartile range). Categorical data is presented as absolute numbers (percentage) of participants. Mann-Whitney U or Chi-squared test were used as appropriate. Abbreviations: ADP – adenosine diphosphate, HbA1c – glycated hemoglobin A1c; HDL – high-density lipoprotein; LDL – low-density lipoprotein; NIHSS – National Institute of Health Stroke Scale; mRS – modified Rankin’ Scale. *p<0.05, **p<0.01, ***p<0.001.
