# Supplementary material for: Associations of sphingosine-1-phosphate with soluble P-selectin and adverse clinical outcome in patients with cerebral ischemia with and without acetylsalicylic acid treatment
Source: Naunyn Schmiedebergs Arch Pharmacol. 2025 Oct 8;399(3):3743–50. doi: 10.1007/s00210-025-04595-w (PMC12935708; doi:10.1007/s00210-025-04595-w)
Supplement: Supplementary file 2 — Supplementary file2 (DOCX 43 KB) [file 210_2025_4595_MOESM2_ESM.docx]

**Patient flow chart of MARK-STROKE**

Screening for eligibility

and blood draw (n = 413)

1. *Suspected* ischemic stroke or transient ischemic attack
2. 18 years or older
3. Written consent

**Screening**

Patients retrospectively excluded (n = 39)

1. Stroke mimics

(n = 39)

Patients meeting inclusion criteria (n = 374)

1. *Confirmed* ischemic stroke or transient ischemic attack
2. 18 years or older
3. Written consent

Inclusion into cross-sectional analyses (n = 374)

**Inclusion**

Patients with loss of follow-up

(n = 100)

Patients with available follow-up

(n = 274)

Inclusion into longitudinal analyses (n = 274)

**Follow-up**
